# Polycystic ovary syndrome negatively affects sexual function and lower urinary tract symptoms in syrian women: a case-control study

**DOI:** 10.1038/s41598-025-85544-8

**Published:** 2025-01-06

**Authors:** Ali Alshiekh, Rana Hadakie, M Fadi Al Kurdi, Luna sukkar, Marwan Alhalabi, Hamoud Hamed

**Affiliations:** 1 Department of Surgery, Faculty of Medicine, Damascus University, Damascus, Syrian Arab Republic; 2 Al-Assad University Hospital, Damascus University, Damascus, Syrian Arab Republic; 3 Department of Biochemistry and Microbiology, Faculty of Pharmacy, Damascus University, Damascus, Syrian Arab Republic; 4 Department of Gastroenterology and Hepatology, Faculty of Medicine, Damascus University, Damascus, Syrian Arab Republic; 5 Division of Reproductive Medicine, Embryology and Genetics, Faculty of Medicine, Damascus University, Damascus, Syrian Arab Republic

**Keywords:** PCOS, Sexual dysfunction, Lower urinary tract symptoms, FSFI, BFLUTS

## Abstract

Polycystic ovary syndrome (PCOS) is the most prevalent endocrine disorder in women of reproductive age worldwide, and its related features like obesity, mental health issues and hyperandrogenism may contribute to inadequately investigated health problems such as sexual dysfunction (SD) and lower urinary tract symptoms (LUTS). Therefore, this study examined the impact of PCOS on sexual function (SF) and lower urinary tract in Syrian women by recruiting a total of 178 women of reproductive age, of whom 88 were diagnosed with PCOS according to the Rotterdam criteria and 90 without PCOS were considered as the control group. Female sexual function index (FSFI) and Bristol Female Lower Urinary Tract Symptom Questionnaire (BFLUTS) were used to assess SF and LUTS respectively. PCOS group had higher SD prevalence compared to control group (65.9% vs 48.9%, *p* = 0.016), and BMI showed an inverse correlation with the total FSFI score in PCOS group (*p* = 0.027, r = -0.235). Furthermore, PCOS group exhibited significantly lower scores in orgasm and satisfaction subdomains. Additionally, PCOS patients had significantly higher total BFLUTS score compared to control group (median 8 vs 5, *p* = 0.025). Thus, PCOS may be related to SD and LUTS, highlighting the importance of evaluating SF and urinary symptoms in PCOS patients.

## Introduction

Polycystic ovary syndrome (PCOS) is a widely prevalent and heterogeneous endocrine disorder that poses a significant public health concern, affecting approximately 5–20% of reproductive-aged females worldwide^1,2^. The diagnosis of PCOS in adults is confirmed when two of the following three Rotterdam criteria are met: oligo-anovulation, clinical or biochemical hyperandrogenism, and polycystic ovarian morphology (PCOM) observed through sonography^1,3^. PCOS is a multifactorial disease with various contributing factors, including genetics, epigenetics, environmental influences, obesity, insulin resistance, and hyperandrogenism^1,4^. Moreover, PCOS has significant negative consequences for women’s health and quality of life. These consequences include excessive hair growth, acne, subfertility, challenges in weight loss, metabolic syndrome, cardiovascular disease, and psychological disorders such as depression, anxiety, and reduced self-esteem^4,5^. Many consequences associated with PCOS, particularly obesity and mental health issues, and the medications used for its management, are shared risk factors for both sexual dysfunction (SD) and lower urinary tract symptoms (LUTS)^6,7^ which are health problems that have been inadequately investigated in PCOS patients. Female sexual dysfunction (FSD) is a commonly underestimated condition that affects approximately 40–50% of women worldwide. It is typically characterized by a lack of sexual desire or difficulties in arousal, lubrication, or achieving orgasm. It may also manifest as pain during sexual activity. The etiology of FSD involves both organic and psychogenic factors, including obesity, psychiatric disorders, and metabolic syndrome, which are comorbidities associated with PCOS^7–9^. Thus, PCOS may be a risk factor for FSD. LUTS encompass symptoms related to urine storage, voiding, and postmicturition, such as urgency, nocturia, urge incontinence, and intermittent stream. The estimated prevalence of LUTS in females ranges from 5 to 70%, indicating a high prevalence of this issue among women, particularly older women^10,11^. It has been reported that obesity, anxiety and medications used for treatment of mental health disorders are associated with LUTS in females^12–14^. Therefore, similar to FSD, PCOS may contribute to the development of LUTS. Furthermore, LUTS can contribute to SD in women^10,12^. Both SD and LUTS negatively impact women’s quality of life, leading to physical, social, and psychological consequences. This underscores the importance of evaluating both SD and LUTS in women^8,10,15^, especially in PCOS patients to improve the management of PCOS and its related health problems. However, reports on the prevalence of FSD in PCOS patients are contradictory, with some studies indicating lower sexual function (SF) in women with PCOS, while others found no significant differences^7,16,17^. Furthermore, there are currently no studies that have evaluated SF in Syrian PCOS patients. Additionally, there is a limited number of studies investigating the relationship between LUTS and PCOS^6,14,18^. Therefore, the objective of the current study is to assess SF and LUTS in Syrian PCOS patients and to explore the relationship between SD and LUTS in this population.

## Material and methods

### Study design and participants

This is a case-control study that was conducted between December 2023 and Apr 2024 at The University Hospital of Obstetrics and Gynecology in Damascus. Inclusion criteria were to be a sexually active woman of reproductive age (18–45 years old) who was diagnosed with PCOS based on the Rotterdam criteria by an expert gynecologist, did not have any other medical conditions such as type 2 diabetes, cancer, or hypertension, and had not undergone pelvic surgery. Participants in the control group needed to be similar to those in PCOS group in all aspects except for the presence of PCOS. Women who were pregnant, had undergone multiple vaginal deliveries, or refused to participate were excluded from the study. The minimum sample size was estimated using G*Power, which calculated that at least 88 participants were required in each group to achieve a 5% error rate and 95% statistical power.

### Ethical aspects

This study was approved by the ethical committee at Damascus University and was conducted in accordance with the principles outlined in the Declaration of Helsinki. Additionally, a written informed consent was obtained from every participant after explaining the aims of the current study and ensuring the privacy of their data.

### Data collection tools

Data were collected using a self-reporting questionnaire that consisted of three separate sections: general characteristics of participants, the Female Sexual Function Index (FSFI), and the Bristol Female Lower Urinary Tract Symptom Questionnaire (BFLUTS).

**General characteristics of participants:** This section included 10 statements about participant characteristics, such as age, educational level, place of residency, and current smoking status. Additionally, the weight and height of each woman were measured upon agreeing to participate in this study, and their body mass index (BMI) was calculated by dividing weight (in kilograms) by the square of height (in meters). Consequently, women were classified according to BMI as follows: underweight (BMI < 18.5), normal weight (18.5 ≤ BMI < 25), overweight (25 ≤ BMI < 30), and obese (BMI > 30)^19^.

**The Female Sexual Function Index (FSFI):** A widely used, valid, and reliable multidimensional self-report questionnaire designed to assess key aspects of female sexual function over the last four weeks. It was first developed by Rosen et al. and consists of 19 items divided into six subscales: sexual desire (two questions), arousal (four questions), lubrication (four questions), orgasm (three questions), satisfaction (three questions), and pain (three questions). The overall score on the FSFI ranges from 2 to 36, with higher scores indicating better sexual function^20^. A cut-off value of 26.55 is considered an indicator of the presence of SD in females^9^. The current study utilized a previously validated Arabic version of this questionnaire^21^. Cronbach’s alpha for FSFI in this study was 0.852, indicating a high internal consistency^22^.

**The Bristol Female Lower Urinary Tract Symptom Questionnaire (BFLUTS):** A valid instrument developed by Jackson et al. that consists of 19 items designed to assess female LUTS. The final score ranges from 0 to 71, with higher scores indicating more severe LUTS and a greater negative impact on sexual and overall quality of life. This tool comprises five sub-dimensions: filling (BFLUTS-IS - four questions), voiding (BFLUTS-VS - three questions), urinary incontinence (BFLUTS-IS - five questions), quality of life (BFLUTS-QoL - five questions) and sexual life (BFLUTS-sex - two questions). No cutoff value has been reported for this tool^23,24^. We did not find a validated Arabic version of the BFLUTS questionnaire. Therefore, we conducted a translation and validation process for the BFLUTS questionnaire as follows:

**(a) Forward Translation**

The English version of the BFLUTS questionnaire was translated into Arabic by two independent bilingual authors fluent in both languages (MFA and MA). both authors are native Arabic speakers. One of the authors possesses expertise in urology and gynecology terminology, while the other had no prior knowledge of the instrument. This process resulted in two distinct Arabic versions of the questionnaire. Subsequently, a third independent translator and the local coordinator (AA), who holds a PhD, along with the two translators, formed an expert panel to compare these translations with the original BFLUTS questionnaire. They identified and resolved any inadequacies in the translation and expressions to ensure cultural relevance and consistency between the original and translated versions. Consequently, a preliminary Arabic version of the BFLUTS questionnaire was produced.

**(b) Back-Translation**

Two healthcare professionals, whose mother tongue is English and who had no prior knowledge of the original BFLUTS, performed a back-translation of the preliminary Arabic version of the BFLUTS questionnaire into English. This process was supported by the expert panel, who had previously reviewed the forward translation., they compared the two back-translation versions with the original and the preliminary Arabic version of the BFLUTS questionnaire to ensure clarity and accuracy in wording and phrasing. Necessary changes were made to the preliminary Arabic version, resulting in a pre-final Arabic version of the BFLUTS questionnaire.

**(c) Pilot Testing**

The pre-final Arabic version was piloted with 20 native Arabic-speaking women. Each participant completed the questionnaire to assess its clarity and comprehensibility, aiming to evaluate the effectiveness of the translation. Following the completion of the BFLUTS questionnaires, each woman was interviewed individually to discuss her responses. Participants were asked to rate the clarity of each question using a dichotomous scale (clear or unclear) and to express their understanding in their own words. They were invited to highlight any words or phrases that were confusing or could lead to misunderstandings. Feedback from these interviews was used to make necessary adjustments to enhance the relevance of the questionnaire for Arabic-speaking women, which led to the final version of the Arabic BFLUTS questionnaire.

**(d) Validity and Reliability Testing**

Face validity was assessed during the pilot study through cognitive interviewing using the dichotomous scale. Content validity was continuously evaluated by the expert panel throughout the translation process. Finally, internal consistency was assessed using Cronbach’s alpha, based on a random sample of 40 women from the same population of the main study. The obtained Cronbach’s alpha value was 0.76, indicating adequate internal consistency^22^.

The finalized Arabic version of the BFLUTS questionnaire was subsequently utilized to study the severity of LUTS in the current study. In this study, the internal consistency of the BFLUTS was again evaluated, yielding a Cronbach’s alpha of 0.70.

### Statistical analysis

SPSS version 25 was employed in the current study to perform the statistical study. Nominal data was presented using frequency and percentage while numerical data by using median and interquartile. Normality of data distribution was tested using One-sample Kolmogorov–Smirnov test. Mann–Whitney U and Kruskal Wallis tests were applied to compare differences between two or more independent groups respectively. presence of differences between categorical variables was analyzed with Chi square test or Fisher’s exact test. Correlation between numerical variables was studied by Pearson or Spearman test according to the distribution of data. ROC curve test was employed to test the possibility of using a variable in predicting the presence of a condition. *p* ≤ 0.05 indicate statistical significance.

## Results

The current study recruited 178 women of reproductive age (18–45 years old), among whom 88 (49.44%) had PCOS that have been diagnosed according to Rotterdam criteria, and 90 (50.56%) without PCOS were considered as the control group. There were no significant differences between the two groups regarding age, BMI, educational level and smoking status (*p* = 0.883, *p* = 0.376, *p* = 0.693, *p* = 0.106 respectively). Among the PCOS group, 43.2% were classified as overweight or obese in contrast to 35.6% in the control group (*p* = 0.12). Menstrual abnormalities were significantly more prevalent in the PCOS group (*p* = 0.001). Additionally, 28.4% of PCOS patients reported infertility issues while 14.4% reported similar problems in the other group (*p* = 0.004). further general characteristics of the study sample are shown in Table 1.

**Table 1:** General characteristics of the study’s sample.

Variable	Without PCOS n = 90	With PCOS n = 88	*p* value
	n (%)	n (%)	
Age: median (interquartile)	29 (27, 32.25)	29 (26, 31)	*p* = 0.883^†^
BMI: median (interquartile)	23.84 (21.54, 27.01)	24.33 (22.05, 26.69)	*p* = 0.376^†^
BMI classification			
Underweight	4 (4.4)	-	0.12^‡^
Normal weight	54 (60)	50 (56.8)	
Overweight	22 (24.4)	31 (35.2)	
Obese	10 (11.2)	7 (8)	
Education level			
High school or less	7 (7.8)	6 (6.8)	0.693^‡^
University student	14 (15.6)	13 (14.8)	
Bachelor’s holder	58 (64.4)	54 (61.4)	
MSc or PhD	11 (12.2)	15 (17)	
Place of residence			
City	83 (92.2)	79 (89.8)	0.379^+^
Countryside	7 (7.8)	9 (10.2)	
Income level			
Low income	2 (2.2)	5 (5.7)	0.686^‡^
Moderate income	20 (22.2)	20 (22.7)	
Good income	59 (65.6)	54 (61.4)	
High income	9 (10)	9 (10.2)	
Smoking status			
Current smoker	37 (41.1)	25 (28.4)	0.106^‡^
Previous smoker (ceasing smoking for more than a month)	4 (4.4)	3 (3.4)	
Passive smoker	22 (24.4)	24 (27.3)	
Never smoked	27 (30)	36 (40.9)	
Do you have children			
Yes	62 (68.9)	49 (55.7)	0.089^+^
No	28 (31.1)	39 (44.3)	
Fertility problems when trying to get pregnant			
Yes	13 (14.4)	25 (28.4)	0.004*^‡^
No	59 (65.6)	36 (40.9)	
Have not planned to get pregnant yet	18 (20)	27 (30.7)	
Period length (days)			
Less than 21	0 (0)	3 (3.4)	0.001*^‡^
Between 21 and 35	90 (100)	69 (78.4)	
More than 35	0 (0)	16 (18.2)	

*p ≤ 0.05 indicates a significant difference.

^†^ Mann–Whitney U test.

^‡^ Chi-square test.

+ Fisher’s exact test.

### Impact of PCOS on SF

A significantly higher prevalence of SD was noticed in the PCOS group (65.9%) compared to the control group (48.9%) (*p* = 0.024), along with a lower overall FSFI score in the PCOS group (*p* = 0.02).

Regarding the subscales of FSFI, no significant differences were found between women with or without PCOS in terms of desire, arousal, lubrication, and pain (*p* = 0.278, *p* = 0.197, *p* = 0.281, and *p* = 0.492 respectively). However, orgasm and satisfaction scores were higher in the control group (*p* = 0.008 and *p* = 0.007 respectively). Table 2 provides further details regarding SF in the study sample.

**Table 2:** FSFI and its subdomains scores of the study’s sample.

	Without PCOS Median (interquartile)	With PCOS Median (interquartile)	p value
Sexual desire	3.6 (3, 4.2)	3 (2.4, 4.2)	0.278^†^
Sexual arousal	4.2 (3.3, 5.18)	4.05 (3, 5.03)	0.197^†^
Lubrication	5.1 (4.2, 5.7)	4.8 (3.9, 5.7)	0.281^†^
Orgasm	4.8 (4, 5.6)	4.4 (3.2, 5.2)	0.008*^†^
Satisfaction	5.2 (4.4, 6)	4.8 (2.9, 5.6)	0.007*^†^
Pain	3.2 (2.8, 4)	3.6 (2.8, 4)	0.492^†^
FSFI score	26.65 (23.43, 29.4)	24.3 (19.75, 27.88)	0.02*^†^
More than 26.55	46 (51.1%)	30 (34.1%)	0.024*^‡^

*p ≤ 0.05 indicates a significant difference.

^†^Mann–Whitney U test.

^‡^ Fisher’s exact test.

### Impact of PCOS on LUTS

The total BFLUTS score and voiding score were significantly higher in PCOS patients (*p* = 0.025 and *p* = 0.045, respectively). However, no statistical differences were found regarding incontinence, filling, quality of life, and sexual life between the two groups (*p* = 0.226, *p* = 0.093, *p* = 0.118, and *p* = 0.168 respectively). Table 3 showes further details on LUTS in the study sample.

**Table 3:** BFLUST and its subdomains scores of the study’s sample.

	Without PCOS Median (interquartile)	With PCOS Median (interquartile)	p value
Filing	3 (1, 4.25)	3 (2, 5)	0.093
Incontinence	0 (0, 2)	1 (0, 2)	0.226
Voiding	0 (0, 1)	0 (0, 2)	0.045*
Quality of life	1 (0. 3)	1 (0, 3.75)	0.118
Sexual life	0 (0, 0)	0 (0, 0)	0.168
Total BFLUTS score	5 (3, 10)	8 (3, 12.75)	0.025*

*p ≤ 0.05 indicates a significant difference.

Mann–Whitney U test was used in this table.

### Relationship between demographics and FSFI

In the PCOS groups, demographics, except for BMI, did not affect the total FSFI score or any of its subscales. It was noticed a significant inverse correlation between BMI and FSFI, as well as desire, arousal and satisfaction scores (*p* = 0.027, r = -0.235;* p* = 0.031, r = -0.230:, *p* = 0.014, r = -0.262: and *p* = 0.005, r = -0.295 respectively).

**Fig. 1:**
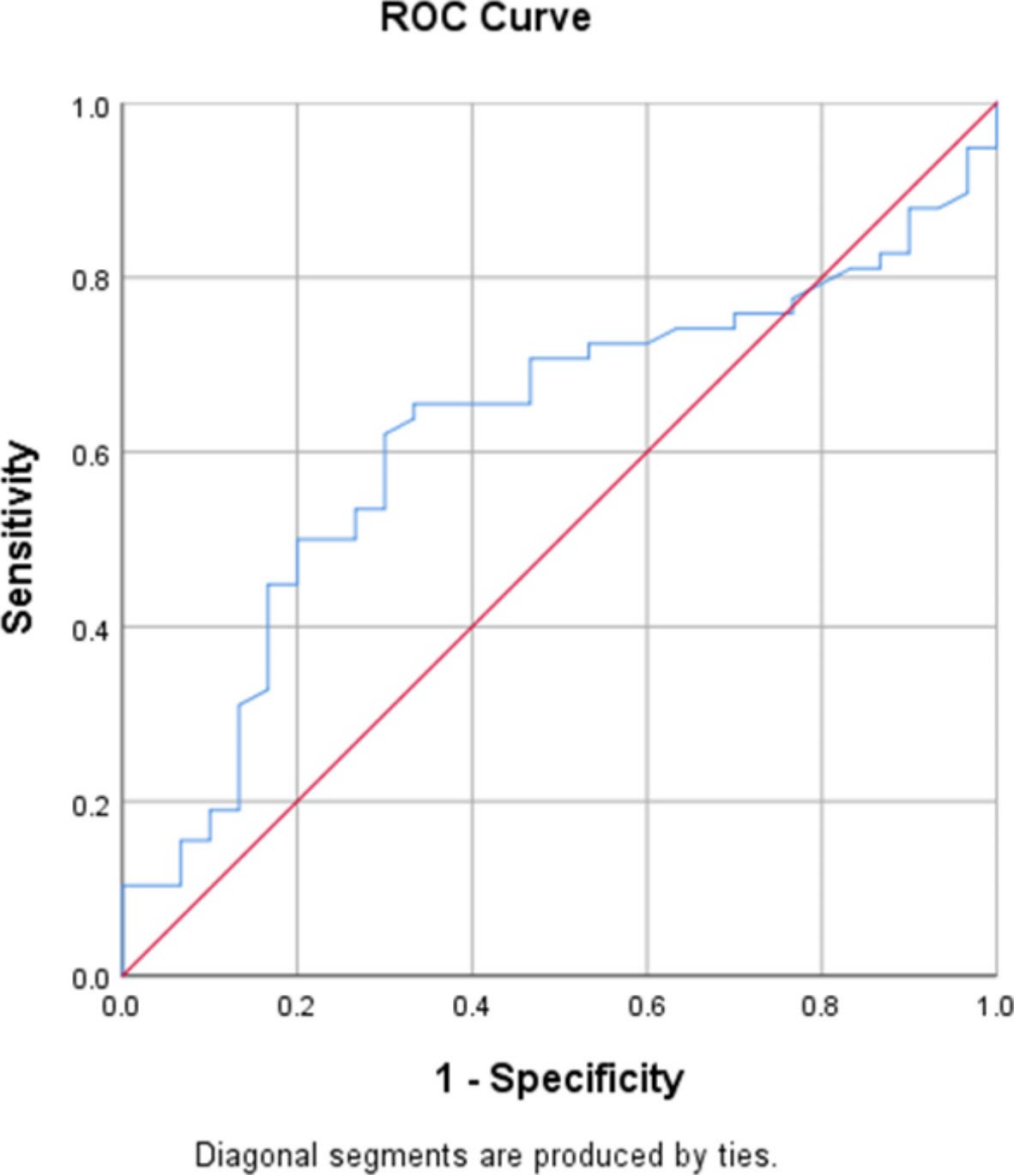
ROC curve of BMI for the prediction of SD in PCOS patients.

Given the relationship between BMI and total FSFI score, ROC curve was employed to test the potential of using BMI to predict the presence of SD in PCOS patients. The area under the curve was 0.621 (Fig. 1). Thus, BMI may serve as a predictive indicator of the existence of SD in PCOS patients, with a cutoff value of 24.16, a sensitivity of 62.1% and a specificity of 70%.

In contrast, the control group, BMI did not correlate with the total FSFI score (*p* = 0.772). However, among other demographics, monthly income was associated with FSFI and sexual desire scores (*p* = 0.029 and *p* = 0.012 respectively). Pairwise comparisons showed higher FSFI and desire scores in the high-income group compared to the low-income group (*p* = 0.029 and *p* = 0.022 respectively). Furthermore, being a current smoker significantly increased pain score compared to never smokers (*p* = 0.047).

### Correlation between demographics and BFLUTS

No relationship was found between BMI and total BFLUTS score in either the PCOS or the control groups (*p* = 0.624 and *p* = 0.379 respectively), nor between BMI and any of the BFLUST subdomains’ scores. Current smoking status affected the incontinence score in the PCOS group (*p* = 0.048), and pairwise comparison showed that current smokers had a higher likelihood of experiencing incontinence compared to never smokers (*p* = 0.044). no statistically differences were found regarding the effect of other demographics on BFLUTS and its subdomain scores in either the PCOS or control groups.

### Correlation between total FSFI and BFLUTS scores

There was no correlation between FSFI and BFLUTS scores in either in the PCOS or control groups (*p* = 0.284 and *p* = 0.576, respectively).

## Discussion

To the best of our knowledge, the current study is the first to evaluate both SF and LUTS in Syrian women with PCOS. Our results revealed a high prevalence of SD among PCOS patients, with about 66% of them having a total FSFI score < 26.55. Additionally, orgasm and satisfaction were significantly affected in PCOS patients compared to controls in our study. Our findings align with those of Mojahed et al., who reported significantly lower scores of total FSFI, lubrication, orgasm, satisfaction, and pain in PCOS patients^17^. Pastoor et al. also found lower total FSFI and all of FSFI subdomains’ scores in women with PCOS in their systematic review and meta-analysis^7^. Similar results were reported by Aba et al.^25^ and Pastoor et al.^15^ who observed lower scores across all FSFI dimensions except for satisfaction and pain, respectively. Conversely, Ercan et al. revealed no significant differences between the PCOS and control groups regarding FSFI subdomains’ scores. However, the relatively small sample size of their study (32 women in each group) may affect the reliability of their results^16^.

PCOS-related features can adversely affect SF in females. While physiological levels of androgens may play a role in maintaining SF, andreduced androgen levels can impair it, some studies suggest that hyperandrogenism which is often associated with PCOS, contributes to the development of SD^12,26^. Furthermore, hyperandrogenism may indirectly affect SF due to its association with hirsutism, acne, and altered body image, which can negatively affect self-esteem, and lead to disrupted SF in PCOS patients^8^.

PCOS-related mental health issues such as depression and anxiety have been reported to negatively impact SF in women with PCOS^17^. Although we did not assess mental well-being in our study, we believe that mental health disorders are closely related to the decline in FSFI score observed in our PCOS sample, as Syrians, especially females, have already experienced stressors imposed by the ongoing Syrian crisis, resulting in a high prevalence of mental health conditions^26^. Therefore, PCOS diagnosis in a female belonging to this mentally vulnerable population may exacerbate the pre-existing psychological disorders or contribute to the onset of new cases, further impairing SF. This may also explain the relatively high prevalence of SD in the control group (about 50%).

While infertility is a stressful experience that can contribute to SD^17,27^, our findings did not reveal a relationship between infertility issues and SD. This align with the results reported by Pastoor et al.^7^. This may be explained by having children after the management of infertility problems may diminish the effect of previous infertility on SF in women with PCOS.

In terms of demographics, we found an inverse correlation between total FSFI score and BMI in the PCOS group but not in the control group. Hence, increased BMI may indirectly disrupt SF in PCOS patients by exacerbating othrer symptoms related to PCOS, such as lowself-steem and reduced body image. Pastoor et al. also did not find a relationship between BMI and FSFI score^7^. Arab women are usually hesitant to discuss their sexual problems with healthcare providers due to cultural and religious reasons. Moreover, even healthcare providers do not discuss such issues with their patients. Thus, sexual problems are underestimated in this population^28,29^. Given therelationship between BMI and total FSFI score in our PCOS group, we set a BMI cutoff value of 24.16, which may predict the presence of SD in PCOS women with a sensitivity of 62.1% and specificity of 70%. However, this value should only be used to predict the presence of SD in PCOS women with a BMI ≥ 24.16, rather than for the diagnosis of this issue in this group. If the prediction of the presence of SD is made, then more accurate diagnostic assessments may be done.

The total BFLUTS score was significantly higher in the PCOS group, indicating a negative impact of PCOS on lower urinary tract, which led to LUTS. This result is supported by Kölükçü et al., who reported higher BFLUTS scores and elevated scores in all of its subdomains among women with PCOS^6^. Although voiding score was significantly higher in the PCOS groups, the scores of filing, incontinence, quality of life and sexual life did not differ between our two groups. Antônio et al. found that PCOS patients showed an absence of urinary incontinence, while the control group exihabated a higher prevalence of this condition^18^. Additionally, Montezuma et al. reported that urinary incontenece was more closely related to obesity not to PCOS itself^14^.

It has been hypothesized that hyperandrogenism in PCOS may be protective against urinary incontinence, potentially due to increased muscle mass and pelvic floor muscle strength^14,18^. However, Antônio et al. reported that pelvic floor muscle strength was higher in their non-obese PCOS sample but without a statistically significance. This suggests that highertestosterone levels in PCOS patients may protect against urinary incontinence by providing better support for the pelvic floor structure, although they may not be sufficient to enhance of pelvic floor muscle strength^18^. furthermore, a study on female rats showed that testosterone-based treatment enhances stress urinary incontinence^30^. Nevertheless, Kölükçü et al. found a relationship between increased testosterone levels and a higher frequency of LUTS^6^.

Although obesity is associated with urinary incontinence^14^, our results showed that BMI did not correlate with the scores of the total BFLUTS and its subdomains, which contradicts previousstudies.^6,14^. we believe that the low percentage of obese participants in our sample may have affected the accuracy of these results. Given that testosterone levels were not measured in the current study, and that BMI did not correlate with LUTS, the higher BFLUTS score among our PCOS patients may be explained by having previous mental health issues, especially anxiety, which may be associated with the experience of LUTS in Syrian PCOS patients, as Syrians are vulnerable to these mental health issues^26^. The current study did not reveal a relationship between SD and LUTS in PCOS patients. This absence may be due to the influence of PCOS-related features, such as low self-esteem, depression, and anxiety, which are more likely to affect SD rather than LUTS.

Our findings have significant implications for both research and clinical practice. Future studies should further investigate the complex relationship between PCOS, SF, and LUTS. The current study has several limitations, including being a single-center study that did not assess hormonal levels such as testosterone. Additionally, we did not evaluate mental health issues, such as depression and anxiety, which may significantly impact SF and LUTS in women with PCOS. One reason for this is the length of the questionnaire (45 questions) which addressed sensitive aspects of women’s lives. Therefore, we aimed to simplify the questionnaire to encourage participants to complete it fully.

## Conclusion

The results of the current study suggest that PCOS negatively affected SF and lower urinary tract, leading to SD and LUTS in Syrian women. Additionally, the total FSFI score is inversely correlated with BMI in PCOS patients, while BMI did not correlate with the total BFLUTS score in those patients. These results emphasize the need for comprehensive evaluations of SF and urinary symptoms in PCOS patients to better management of PCOS and its related consequences.

## Data Availability

The datasets used and analyzed during the current study are available from the corresponding author on reasonable request.

## Abbreviations

BMI: Body mass index
BFLUTS: Female Lower Urinary Tract Symptom Questionnaire
FSD: Female sexual dysfunction
FSFI: Female sexual function index
LUTS: Lower urinary tract symptom
PCOS: Polycystic ovary syndrome
SD: Sexual dysfunction
SF: Sexual function
